# Fecal melatonin as a biomarker of emerging circadian maturity and gut microbiota in infancy

**DOI:** 10.1038/s44323-026-00080-6

**Published:** 2026-04-28

**Authors:** Mohammed Al-Andoli, Petra Zimmermann, Sarah Schoch, Andjela Markovic, Christophe Mühlematter, Matthieu Beaugrand, Oskar G. Jenni, Rabia Liamlahi, Jean-Claude Walser, Dennis Nielsen, Salome Kurth

**Affiliations:** 1 Department of Psychology, University of Fribourg, Fribourg, Switzerland; 2 Department of Community Health, Faculty of Science and Medicine, University of Fribourg, Fribourg, Switzerland; 3 Faculty of Health Science and Medicine, University of Lucerne, Lucerne, Switzerland; 4 Department of Pulmonology, University Hospital Zurich, Zurich, Switzerland; 5 Donders Institute for Brain, Cognition and Behaviour, Radboud University Medical Centre, Nijmegen, the Netherlands; 6 Translational Research Center, University Hospital of Psychiatry and Psychotherapy, Bern, Switzerland; 7 University Children’s Hospital Zurich, Zurich, Switzerland; 8 University of Zurich, Zurich, Switzerland; 9 Genetic Diversity Center, ETH Zurich, Zurich, Switzerland; 10 Department of Food Science, University of Copenhagen, Copenhagen, Denmark

**Keywords:** Gastroenterology, Microbiology, Neuroscience

## Abstract

Melatonin plays a key role in circadian regulation, and its interaction with the gut microbiota may be critical for early-life development. Beyond its circadian function, melatonin dysregulation is implicated in inflammatory, metabolic, psychiatric, and neurological disorders. While the gastrointestinal tract produces melatonin at levels far exceeding the pineal gland, its role in gut microbiota dynamics and circadian maturation remains unclear. This observational study investigates the association between fecal melatonin levels and microbial diversity, specific bacterial taxa (ZOTUs), actimetry-based sleep metrics, and various time-dependent factors (including *stool timing* and *intervals since the last stool, sleep, and meal*) in infants at 3, 6, and 12 months of age. Key findings include: (1) fecal melatonin levels increase with age but show high inter-individual variability; (2) fecal melatonin is associated with time factors, such as *stool timing* and *time since last stool*; (3) higher fecal melatonin levels are linked with reduced gut microbial richness and diversity; (4) the number of bacterial taxa associated with fecal melatonin decline over time; (5) melatonin is associated with age-dependent shifts in both bacterial phyla and genera, notably increasing phyla *Actinobacteriota* and *Bacteroidota*, and genera Bifidobacterium, and *Veillonella*, while reducing the phylum Firmicutes and the genus Streptococcus; (6) fecal melatonin is linked to circadian maturation; and (7) finally, *stool timing* variability and *fasting time* affect fecal melatonin stability. These findings identify intestinal melatonin as a promising biomarker for gut microbiota development and circadian rhythm establishment. In addition, these insights highlight melatonin in infant stool as a biomarker and potential modulator bridging systems of intestinal microbiota and behavioral sleep-wake organization.

## Introduction

Melatonin (N-acetyl-5-methoxytryptamine) is one of the most conserved biological molecules and a ubiquitous hormone found across diverse life forms. Initially serving as an antioxidant in unicellular organisms^1,2^, its biosynthetic origins likely emerged from bacterial endosymbiosis, with *Rhodospirillum rubrum* identified as an early producer of melatonin^3^. Melatonin evolved into a signaling molecule in multicellular organisms, linking light-dark cycles to endocrine and physiological processes^4^. In humans, melatonin peaks during the early night to promote sleep and stays low during periods with (blue) light exposure^5^, while its production in the intestines suggests potential systemic effects beyond its pineal-derived functions^6^.

Melatonin is primarily released by the pineal gland in mammals, including humans, during darkness. Light in the frequency (440–480 nm)^7^ activates retinal neurons, which relay signals to the hypothalamic suprachiasmatic nucleus, suppressing melatonin release and aligning biological timing with environmental light, thereby impacting alertness. The suprachiasmatic nucleus also integrates hormonal signals with serotonin, the biochemical precursor of melatonin, regulating the circadian phase and sleep initiation. Seasonal variation and additional systemic roles of melatonin have been reviewed elsewhere^8,9^.

Traditionally, melatonin research has focused on its presence in blood, saliva, or urine to understand its systemic roles in circadian regulation and sleep behavior^10^. However, recent findings highlight the gastrointestinal tract as a major site of melatonin production, where levels can exceed those of the pineal gland by up to 400-fold^7^. Unlike pineal-derived melatonin, gut-derived melatonin is released independently of light and acts locally on gastrointestinal motility, immune function, and interactions with the gut microbiome^11,12^.

The gut microbiome, composed of trillions of microorganisms that reside in the gastrointestinal tract, plays a central role in infant health, particularly in digestion, immune system development, and overall health and development^13^. During the first year of life, the infant gut undergoes rapid colonization, with microbial diversity and composition evolving in response to factors, such as diet, household composition, lifestyle, urban/rural context, and sleep^14,15^. Given the local production of melatonin in the gut, bidirectional interactions between melatonin and the microbiota are likely, whereby melatonin may shape microbial composition and microbial activity may influence melatonin metabolism^8^. These interactions may be particularly relevant during infancy, a critical period for microbiota establishment and circadian maturation^15^.

Melatonin is essential for infants, particularly in early life when endogenous production is still developing, and they partially rely on maternally derived melatonin via breast milk, which exhibits circadian fluctuations^16^. Melatonin plays a role in sleep regulation, neurodevelopment, and oxidative stress mitigation, and melatonin administration can alleviate infantile colic^16,17^. While previous studies have focused on salivary or urinary melatonin quantification^18,19^, fecal melatonin patterns remain obscure, and may yet provide complementary insight into systemic infant melatonin dynamics.

Emerging evidence reveals rhythmic patterns in rodent and infant gut microbiota composition^20,21^ and suggests that microbial rhythmicity is linked to sleep-wake cycles and circadian maturation^22^. Time-sensitive factors, such as stool-timing, time since last bowel movement, sleep, and feeding may therefore modulate fecal melatonin levels and their association with gut microbiota and circadian development^20,23,24^. In line with this, fecal melatonin has further been linked to behavioral and physiological processes related to circadian regulation and feeding behavior^12,25^. Sleep-wake patterns, which are influenced by melatonin^26,27^, can be objectively assessed using actimetry, and salivary melatonin rhythms closely align with actimetry-derived sleep and circadian markers^28^. However, no studies have systematically investigated how these time-based factors relate to fecal melatonin during infancy.

This study addresses this gap by characterizing the relationships among fecal melatonin levels, gut microbiome composition, sleep-wake organization, and time-based factors in infants at 3, 6, and 12 months of age. We hypothesized that (i) fecal melatonin concentrations vary with time-of-sampling factors (stool timing, time since last stool, sleep, and feeding); (ii) fecal melatonin is associated with gut microbiota composition and microbial rhythmicity; and (iii) fecal melatonin relates to circadian maturation, reflected in actimetry-derived measures, such as the Circadian Function Index (CFI).

## Results

Melatonin levels in infant stool samples exhibited a mean concentration of 163.40 pg/g across all age groups, with values ranging widely from 24.56 pg/g to 995.73 pg/g (Table 1). In addition, data demonstrate sample variability of stool melatonin concentration through SD values, which show Inter-individual variability in fecal melatonin concentrations was highest at 3 months (SD = 150.83), lowest at 6 months (SD = 120.78), and intermediate at 12 months (SD = 131.66). Post hoc Tukey comparisons revealed a borderline increase in melatonin levels at 12 months compared to 3 months (diff = 0.0375, *p* = 0.0544), while no significant differences were found between other time points. These results suggest a potential trend toward increasing melatonin levels with age.

**Table 1:** Melatonin concentration in infant stool for pooled samples and separated by age group.

Age (month)	Mean (pg/g)	SD (pg/g)	Min (pg/g)	Max (pg/g)	*n* (samples)
First year of life (3–12)	185.8	133.33	24.56	995.73	462
3	178.67	150.83	24.56	995.73	151
6	180.71	120.78	25.54	747.25	172
12	198	131.66	31.21	943.08	139

### Associations between melatonin levels and time-based variables

**Fig. 1:**
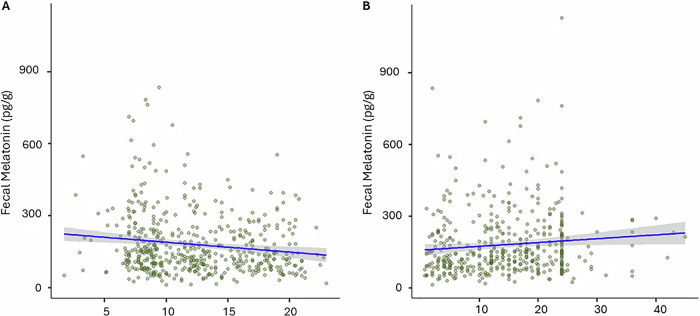
Association between fecal melatonin and time-based factors using mixed models all ages from all cohorts (*n* = 184 subjects, *n* = 486 samples, 54% male, 46% female). **A** *Stool timing* (clock time of collection, i.e., 0 = midnight, 24-h format) shows a weak but statistically robust negative association with fecal melatonin (coef = -0.17, std = 0.05, FDR = 3.8e-5, *r* = −0.16), indicating lower melatonin in stool collected later in the day. **B** *Time since last stool* exhibits a weak but robust positive association with stool melatonin concentration (coef = 0.0825, std = 0.046, FDR = 0.079, *r* = 0.13). This suggests that longer intervals since the last bowel movement correspond to higher melatonin concentration in the fecal sample.

First, we analyzed whether stool melatonin concentration is affected by time-based factors (i.e., *time since last stool*, *stool timing*, s*leep pressure*, *fasting duration*). To account for the repeated measures from the same infants across age groups, we applied mixed models, following a longitudinal approach in MaAsLin2. The strongest effect emerged for *stool timing*, which exhibited a significant negative association with fecal melatonin levels - later stool collection times were linked to lower melatonin (coef = −0.17, std = 0.05, FDR = 3.8e-5, *r* = −0.16; Fig. 1A). In contrast, *time since last stool* showed a weaker positive and non-significant association, indicating that longer intervals between bowel movements were linked to increased fecal melatonin (coef = 0.0825, std = 0.046, FDR = 0.079, *r* = 0.13; Fig. 1B). This effect was consistent across pooled samples from all age groups. No significant associations were found between fecal melatonin and *sleep pressure* or *fasting time*, indicating that these factors do not determine fecal melatonin content in infancy.

We subsequently investigated age-specific effects (Table 2). Consistent with the pooled sample analysis, *stool timing* showed a negative association with fecal melatonin at 3 and 6 months, indicating that samples collected earlier in the day contained higher melatonin concentrations (Fig. 1A). Interestingly, this effect diminished by 12 months.

**Table 2:** Multivariable associations between fecal melatonin and time-based factors across three age groups (3, 6, and 12 months).

	3 months				6 months				12 months			
Time-based measure	coef	std	FDR	*r*	coef	std	FDR	*r*	coef	std	FDR	*r*
*Stool timing*	−0.23	0.08	0.005	−0.26	−0.16	0.07	0.02	−0.2	−0.11	0.07	0.15	−0.1
*Time since last stool*	0.16	0.1	0.1	0.19	0.14	0.08	0.06	0.17	0.03	0.08	0.72	0.02
*Sleep pressure*	−0.1	0.09	0.28	−0.09	−0.03	0.08	0.67	−0.03	−0.04	0.08	0.6	−0.04
*Fasting time*	0.1	0.13	0.44	0.06	−0.01	0.13	0.94	−0.03	0.09	0.11	0.45	0.02

For *time since last stool*, a positive association with fecal melatonin was observed at 6 months, suggesting that longer intervals between bowel movements, which followed the same positive pattern seen across all ages (Fig. 1B). In contrast, no significant associations were observed between fecal melatonin and *sleep pressure* or *fasting time* at any age (Table 2).

These findings emphasize the effect of two key time-based factors on stool melatonin: sampling time and immediate history of bowel activity. Interestingly, immediate sleep history and feeding history appear to have no measurable impact on infant fecal melatonin concentration.

### Associations between melatonin and gut microbiome composition

**Fig. 2:**
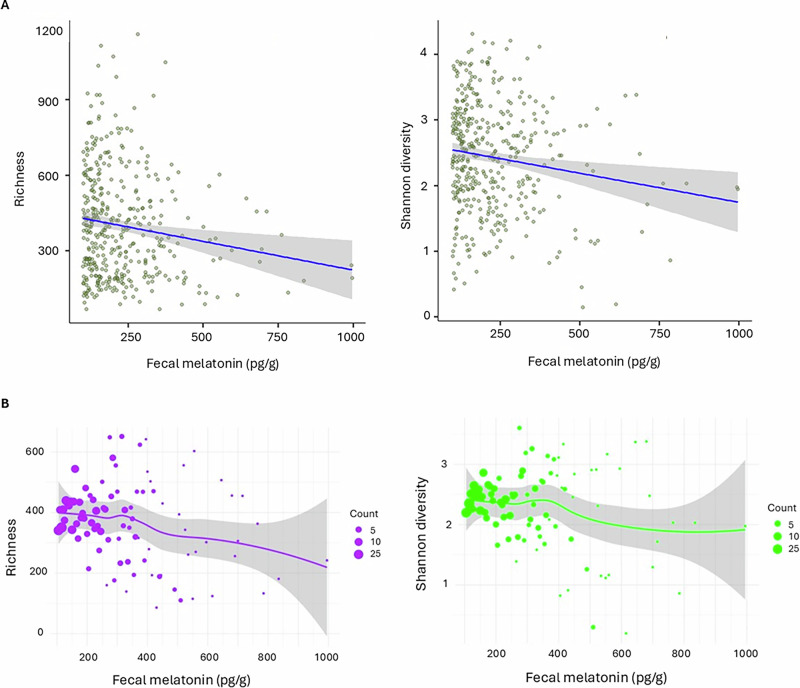
Association of infant fecal melatonin with gut microbial parameters (richness, diversity). **A** Multivariate mixed-effects regression analysis showing the association between fecal melatonin concentration and gut microbial richness (FDR = 0.01) and diversity (Shannon; FDR = 0.006) in infants. Blue lines indicate model-predicted trends for microbiome parameters, adjusted for the clock time of stool sampling. Richness was defined as Observed ZOTUs, representing the total number of detected taxa per sample. **B** Relationship of fecal melatonin and gut microbial parameters, adjusted for improved visual representation. Scatter plots depict individual samples grouped according to equal melatonin concentration. Circle size represents the number of samples combined within a concentration bin, with “count” referring to the number of samples (e.g., samples pooled for melatonin within 100–105 pg/g).

We next examined the relationship between fecal melatonin and key gut microbiota composition parameters (diversity, evenness, richness, relative abundance) in infants aged 3, 6, and 12 months. Using MaAsLin2 for multivariate mixed-model analysis on pooled samples. Figure 2A presents the multivariate model estimates when all infants are analyzed together (e.g., 3,6, and 12 months), while Fig. 2B illustrates the underlying sample distribution for context. We found that the melatonin level was negatively associated with microbial richness (coef = −0.12, std = 0.032, *p* = 0.002, FDR = 0.01, *r* = −0.15), indicating that higher fecal melatonin levels correspond to reduced microbial richness. A similar negative association was observed for microbial diversity (Shannon Index; coef = −0.11, std = 0.040, *p* = 0.0008, FDR = 0.006, *r* = −0.13). No significant associations were found for evenness or relative abundance.

Next, we examined the association between fecal melatonin and microbiome composition for the three age groups: 3, 6, and 12 months (Table 3). Significant effects were found between stool melatonin concentration and gut microbiome composition at the age of 12 months: Richness showed a negative association (coef = −0.074, *p* = 0.018, FDR = 0.08, *r* = −0.2), suggesting reduced microbial richness with higher melatonin levels. Similarly, Shannon diversity and evenness exhibited a weak negative association (Shannon: coef = −0.088, *p* = 0.037, FDR = 0.11, *r* = −21, evenness: coef = −0.056, *p* = 0.04, FDR = 0.12, *r* = −0.17) at this age. No significant associations were observed for fecal melatonin with gut microbacterial evenness or relative abundance across any age group. These findings indicate that melatonin is associated with microbial composition, particularly richness and diversity, as infants approach 12 months.

**Table 3:** Multivariable association between stool melatonin concentration and infant gut microbiome composition across three age groups (3, 6, and 12 months).

*Measure*	3 months				*r*	6 months				*r*	12 months				*r*
	coef	std	*p*	FDR		coef	std	*p*	FDR		coef	std	*p*	FDR	
Richness	−0.118	0.067	0.083	0.593	−0.062	−0.104	0.052	0.048	0.384	−0.19	−0.074	0.031	0.018	0.08	−0.196
Diversity (Shannon)	0.047	0.045	0.297	0.593	−0.131	0.065	0.047	0.17	0.678	−0.065	−0.088	0.042	0.037	0.11	−0.207
Evenness	−0.027	0.06	0.652	0.745	0.097	−0.019	0.043	0.661	0.955	−0.038	−0.056	0.027	0.044	0.118	−0.17
Relative abundance	−0.03	0.062	0.627	0.745	−0.072	−0.016	0.035	0.656	0.955	0.121	−0.012	0.065	0.853	0.853	−0.045

### Associations between stool melatonin, gut microbiota composition, and age-related trends

Next, we refined the analyses to characterize a potential association between stool melatonin and gut microbiota at taxonomic levels, including ZOTU and bacterial phylum relative abundances. Exploratory taxon-level analysis was performed separately within each age group using Pearson correlations between the relative abundance of individual ZOTUs and fecal melatonin concentration.

**Fig. 3:**
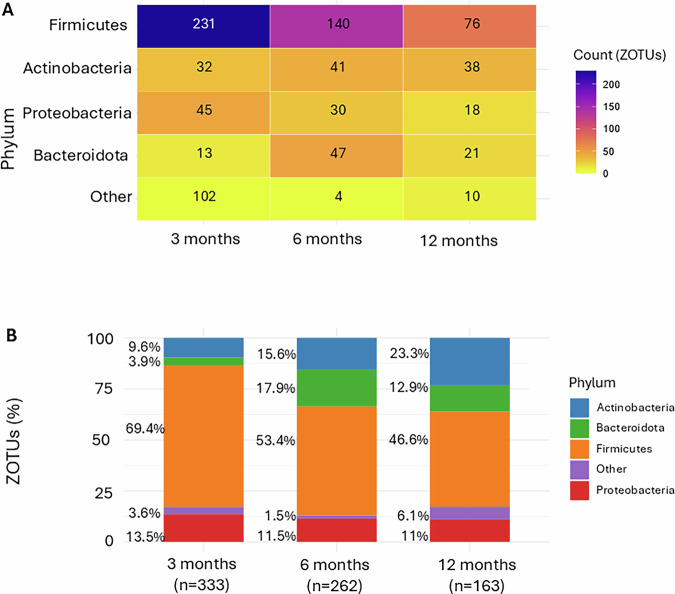
Phylum-level distribution of zotus is positively associated with fecal melatonin across age groups. **A** Heatmap showing the distribution of 758 ZOTUs significantly positively associated with fecal melatonin (*p* < 0.05) across age groups, categorized by bacterial phylum, quantified as ZOTU counts. **B** Proportion of positively melatonin-associated ZOTUs within each phylum at different ages. The total number of positively melatonin-associated ZOTUs per age group is indicated (*n* = 333, *n* = 262, *n* = 163). *Firmicutes* consistently represent the dominant phylum, while *Actinobacteria* become increasingly prominent with age.

**Fig. 4:**
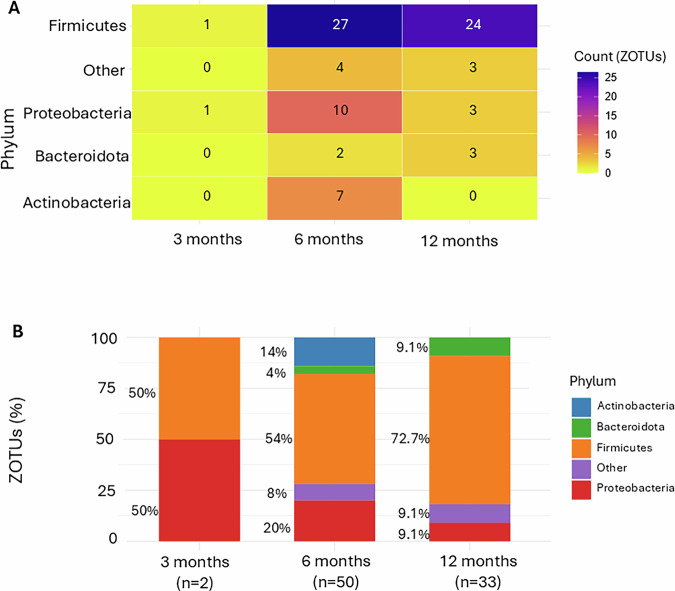
Phylum-level distribution of zotus is negatively associated with fecal melatonin across age groups. **A** Heatmap showing the distribution of 85 ZOTUs significantly negatively associated with fecal melatonin (*p* < 0.05) across age groups, categorized by bacterial phylum, quantified as ZOTU counts. **B** Proportion of negatively melatonin-associated ZOTU within each phylum at different ages. The total number of negatively melatonin-associated ZOTUs per age group is indicated (*n* = 2, *n* = 50, *n* = 33). Firmicutes represent the most dominantly associated phylum, followed by Proteobacteria.

Overall, stool melatonin concentration exhibited significant associations with 843 out of 5735 ZOTUS across age groups (Figs. 3 and 4). Notably, the number of ZOTUs that were associated with melatonin declined with age, with 335 at 3 months, 295 at 6 months, and 183 at 12 months. Thus, a smaller subset of the gut microbiota remains linked to fecal melatonin concentration as infants mature.

Most (758) ZOTUs showed positive relationships with fecal melatonin, with 333 at 3 months, 262 at 6 months, and 163 at 12 months (Fig. 3). Among these, *Firmicutes* was the most strongly associated phylum across all ages, though its relative contribution decreased with age, accounting for 69.4% of positive associations at 3 months, 53.4% at 6 months, and 46.6% at 12 months. In contrast, *Actinobacteria* became increasingly apparent as a prominent link in older infants. By 6 and 12 months, *Bacteroidota* and *Actinobacteriota* emerged as the second most positively associated phyla.

In addition, 85 ZOTUs exhibited negative associations with stool melatonin, indicating that higher melatonin levels were linked to a reduced abundance of these taxa (Fig. 4). *Firmicutes* remained the most frequently associated phylum across all ages. At 6 months, *Proteobacteria* was the second most frequent negatively associated phylum, but by 12 months, this shifted to *Bacteroidota*, indicating an age-related transition in the relationship of microbial phyla with fecal melatonin.

**Fig. 5:**
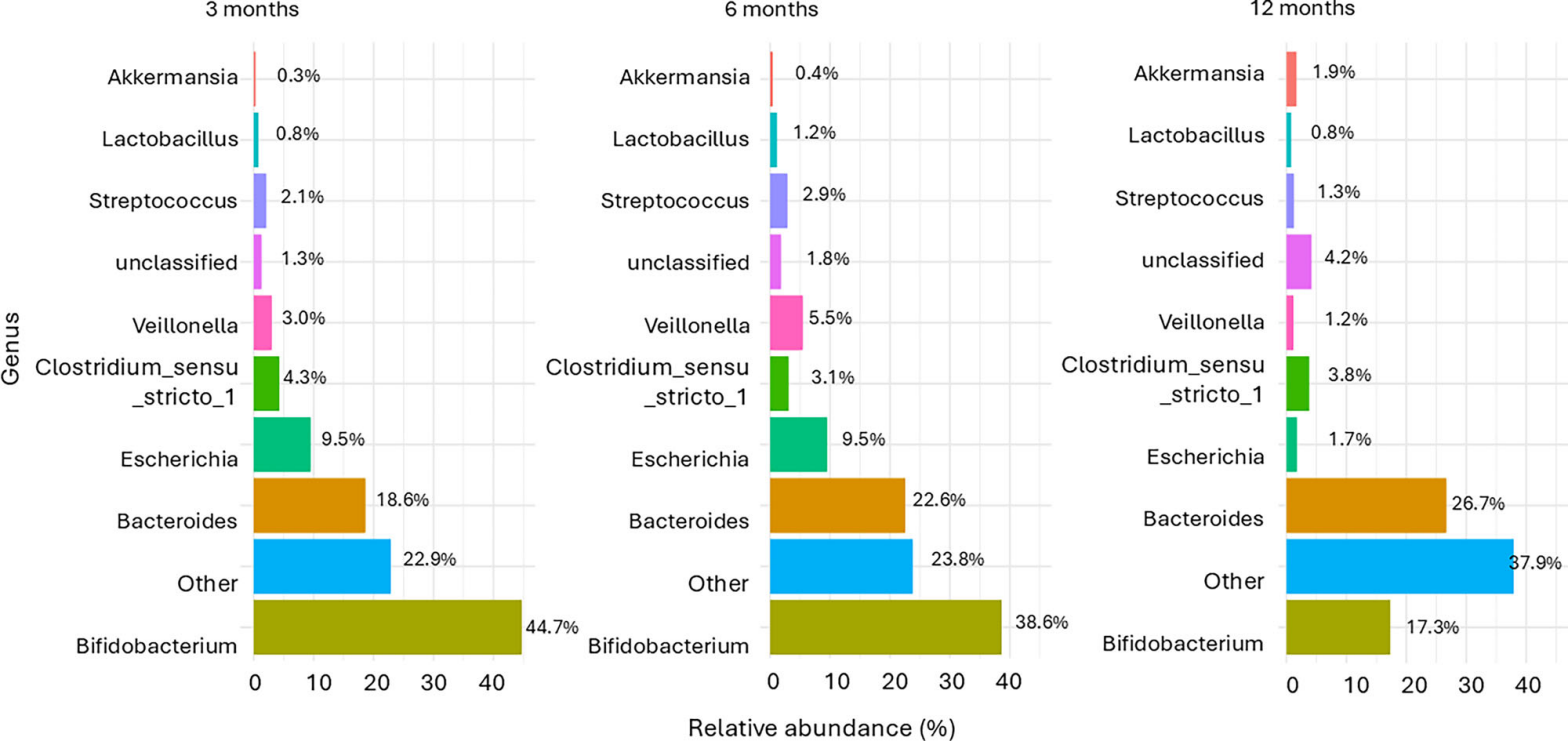
Gut microbial composition at the genus level. The top nine genera are displayed by relative abundance; all remaining, less abundant genera are grouped under “Others.” *Bifidobacterium* represents the most dominantly genus, followed by Bacteroides.

We extended the association analysis from the phylum to the genus level, focusing on relationships between fecal melatonin and bacterial genera. The analysis included the nine most abundant genera, with approximately 180 less prevalent taxa grouped as “Others” (Fig. 5). Relative abundances at 3, 6, and 12 months illustrate age-related shifts in microbial composition. At 3 months, *Bifidobacterium* dominates. By 6 months increases in *Bacteroides*, *Streptococcus*, and *Veillonella* exist. At 12 months, genera like *Akkermansia* and Clostridium_sensu_stricto_1 become more prominent.

**Fig. 6:**
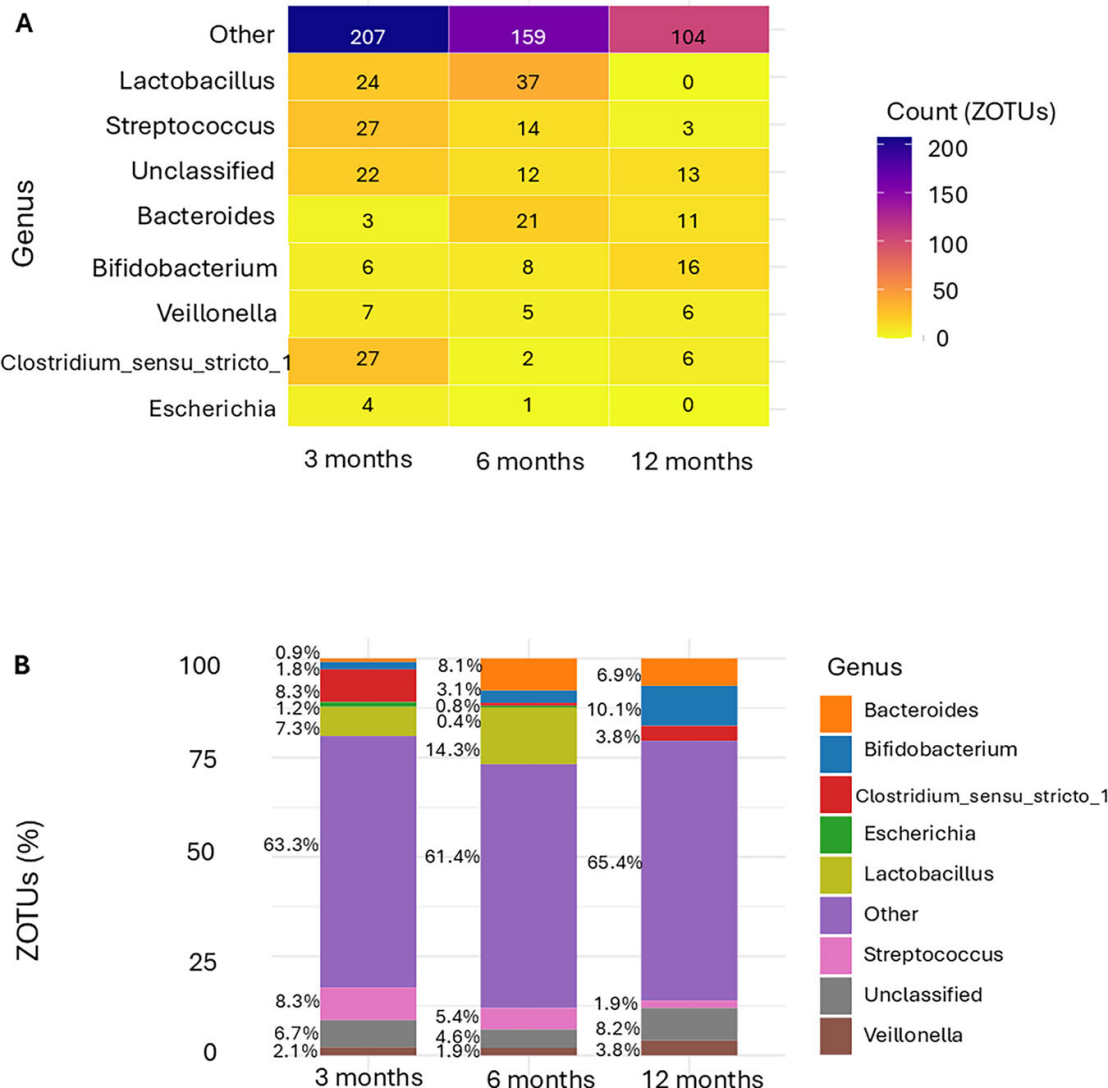
Genus-level distribution of ZOTUs positively associated with fecal melatonin across age groups. **A** Heatmap displaying which 745 ZOTUs were significantly positively associated with fecal melatonin (*p* < 0.05), grouped by bacterial genus, and stratified by infant age. ZOTU abundance is quantified as counts. **B** Proportion of positively melatonin-associated ZOTUs within each genus across age groups. The total number of ZOTUs per age group is indicated (*n* = 327, *n* = 259, *n* = 159). The “Other” category consistently represents the dominant group, while Bifidobacterium becomes increasingly prominent with age.

Most ZOTUs (745) were positively associated with fecal melatonin, with 327 identified at 3 months, 259 at 6 months, and 159 at 12 months (Fig. 6), indicating a decline in the number of significant associations with age. Across all time points, the “Other” category remained the most dominant, suggesting that many melatonin-associated ZOTUs belong to low-abundance genera. Notably, *Bifidobacterium* and *Veillonella* exhibited increasing relative abundance over time, while *Streptococcus* declined. *Lactobacillus* was present at 3 and 6 months but was undetectable by 12 months.

**Fig. 7:**
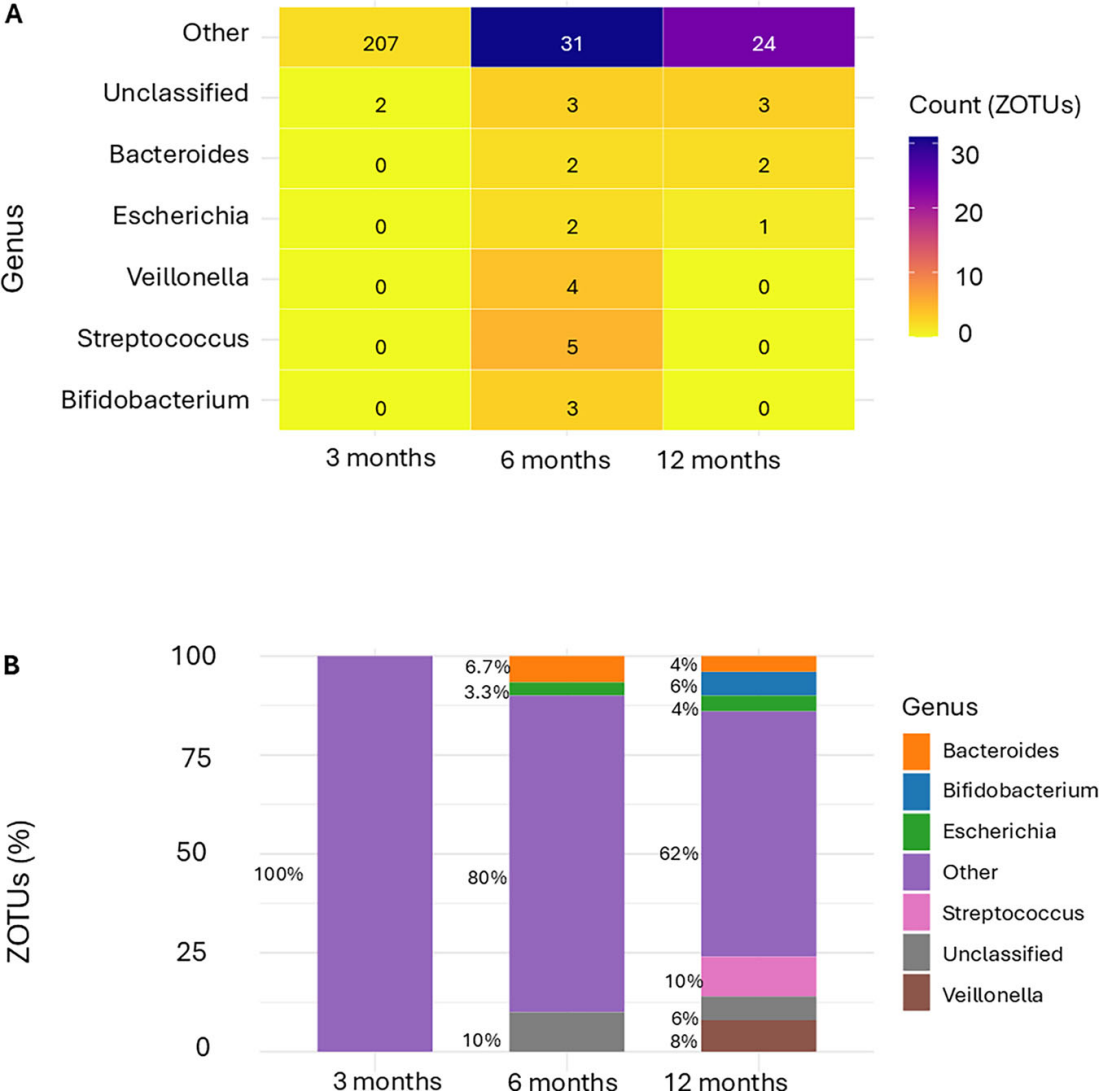
Genus-level distribution of ZOTUs negatively associated with fecal melatonin across age groups. **A** Heatmap showing the distribution of 82 ZOTUs significantly negatively associated with fecal melatonin (*p* < 0.05) grouped by bacterial genus and stratified by age. ZOTU abundance is presented as counts. **B** Proportion of negatively melatonin-associated ZOTU within each genus across age groups. The total number of negatively associated ZOTUs per age group is shown (*n* = 2, *n* = 50, *n* = 33). Firmicutes was the most frequently represented phylum, followed by Proteobacteria.

Conversely, 82 ZOTUs exhibited negative associations with fecal melatonin (Fig. 7), indicating that higher melatonin levels were linked to reduced abundance of these taxa. Among them, Bacteroides (and unclassified genera, i.e., taxa labeled as “uncultured” that could not be confidently assigned to a known genus) became slightly more prominent with age.

We analyzed 360 samples spanning 3, 6, and 12 months and found that total ZOTU richness (i.e., the number of detected ZOTUs) increased substantially with age, from 26,692 at 3 months to 72,261 at 12 months, reflecting gut microbiota maturation. In contrast, the subset of ZOTUs associated with stool melatonin decreased over the same period. We speculate that this divergence may indicate that, while microbial diversity broadens with age, the relationship between fecal melatonin and the gut microbiota narrows to a smaller, potentially functionally relevant subset of taxa.

### Age-related differences in gut microbial community structure and diversity

Our analyses revealed clear developmental changes in alpha diversity across infancy (Supplementary Fig 1). Both Shannon and Simpson indices increased steadily from 3 to 12 months (Kruskal–Wallis, *p* < 2.2 × 10^⁻16^), in line with prior reports that diversity expands as infants transition through major dietary and environmental stages (Yatsunenko et al.^29,30^). Richness also rose markedly with age, suggesting the continual acquisition and establishment of new taxa, while evenness improved, indicating a more balanced microbial distribution by 12 months. Together, these findings point to a robust maturation of the gut microbiome between 3, 6, and 12 months, independent of any relationship with melatonin. Pairwise comparisons (Dunn’s test with Bonferroni correction) showed significant differences between all age groups for Shannon diversity, Simpson diversity, observed richness, and evenness, except for evenness between 3 and 6 months, which was not significant (Supplementary Fig. 1).

Beta diversity analyses supported these developmental patterns. The PCoA based on Bray–Curtis dissimilarity (Supplementary Fig. 2) revealed a clear age-related gradient: samples from 3 months clustered tightly and distinctly, showing lower dispersion, whereas those from 6 and especially 12 months were more broadly distributed across ordination space. This widening spread at older ages suggests increasing inter-individual variability, a feature commonly associated with microbiome diversification during later infancy. The separation between age groups along PC1 and PC2 reflects progressive shifts in community composition over time. Permanova confirmed these age-related differences, with age explaining 5.19% of the variance in Bray–Curtis distances (*R*² = 0.0519, *p* = 0.001) and 7.31% of variance in Aitchison distances (*R*² = 0.0731, *p* = 0.001). Although modest in magnitude, these effects were statistically robust and consistent across dissimilarity metrics.

### Association of infant stool melatonin with sleep onset latency

Next, we examined whether stool melatonin is associated with infant sleep by four BISQ variables: *sleep onset latency*, *nighttime sleep duration*, *night awakenings*, and *daytime sleep duration*. We performed multivariate mixed models analyses to assess these relationships, including *stool timing* as a covariate as it showed that later collection of stool times is strongly associated with lower fecal melatonin levels (FDR = 3.8e-5), making it an essential potential confounder. The results showed a positive association between fecal melatonin and *sleep onset latency* (coef = 0.1096, *p* = 0.02, std= 0.05), which remained marginally significant after multiple comparison adjustment (FDR = 0.053). No significant associations were observed for the other sleep variables (*night awakenings* coef =0.04, std = 0.05, *p* = 0.36, FDR = 0.61; *nighttime sleep duration* coef = −0.01, std=0.05, *p* = 0.83, FDR = 0.86; *daytime sleep duration* coef = −0.01, std=0.05, *p* = 0.84, FDR = 0.86).

Subsequently, we screened for age-specific effects between fecal melatonin and sleep parameters (Table 4). At 3 months, no associations were observed between melatonin and any actimetry-derived sleep variable. However, at 6 and 12 months, *sleep onset latency* exhibited a weak trend-level relationship with fecal melatonin, though this did not withstand FDR correction. No relationships were identified for the remaining sleep parameters.

**Table 4:** Association between fecal melatonin concentration and actimetry-based sleep parameters across three age groups (3 months: n = 127, 6 months: *n* = 134, and 12 months: *n* = 132).

Sleep variable	3 months				6 months				12 months			
	coef	std	*p*	FDR	coef	std	*p*	FDR	coef	std	*p*	FDR
*Sleep Latency*	0.09	0.09	0.32	0.65	0.13	0.09	*0.09*	0.14	0.17	0.08	*0.06*	0.11
*Nighttime sleep duration*	−0.05	0.09	0.4	0.65	0.06	0.09	0.51	0.85	0.11	0.08	0.2	0.34
*Night awakenings*	0.04	0.09	0.65	0.65	0.01	0.09	0.93	0.93	0.03	0.08	0.76	0.76
*Daytime sleep duration*	0.06	0.09	0.53	0.65	0.01	0.09	0.89	0.93	−0.04	0.08	0.62	0.76

Stool timing was used as a covariate variable.

### CFI association with melatonin levels emerging in later-stage infancy

**Fig. 8:**
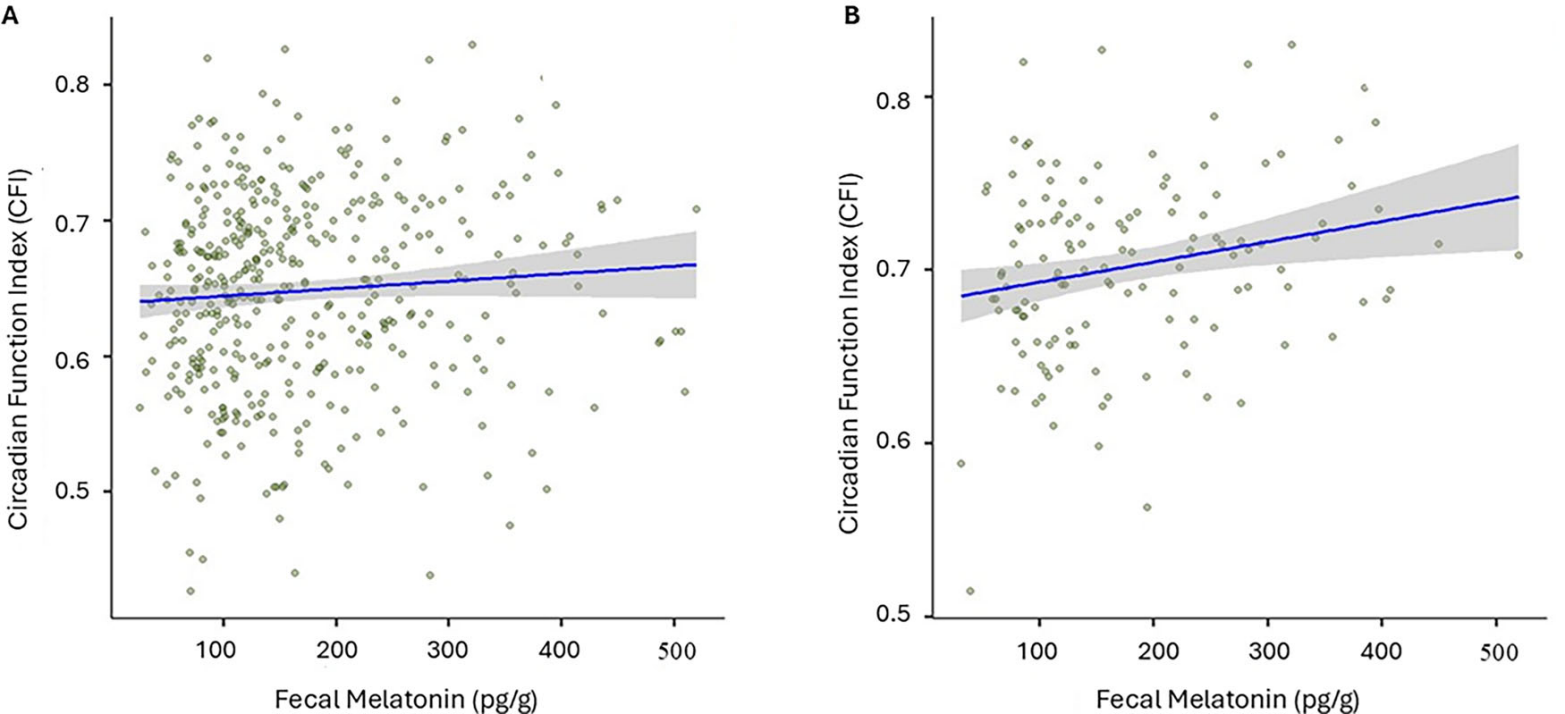
Associations between fecal melatonin levels and CFI. **A** Multivariate mixed-effects model across all ages (*n* = 153 subjects, *n* = 424 samples; coef = 0.013, std = 0.08, FDR = 0.22, *r* = 0.076). **B** Multivariate linear model in 12-month-olds only, with stool timing as a covariate (coef = 0.26, std = 0.07, FDR = 0.01, *r* = 0.235).

To examine a relationship between fecal melatonin and circadian rhythm development, we applied a multivariate mixed-effects model that accounted for repeated measures within infants and included *stool timing* as a covariate, because it strongly predicts lower fecal melatonin levels (FDR = 3.8e-5, as shown in “Associations between melatonin levels and time-based variables"). When analyzing all ages combined, no significant association was found between melatonin and CFI (coef = 0.013, std = 0.08, *p* = 0.08, FDR = 0.22, *r* = 0.076; Fig. 8A). Age-stratified analysis using separate linear models revealed no associations at 3 and 6 months (3 months: coef = −0.021, std = 0.08, FDR = 0.78, *r* = −0.013; 6 months: coef = −0.034, std = 0.07, FDR = 0.63, *r* = −0.05). However, at 12 months, a significant positive relationship with moderate effect size emerged (coef = 0.26, std = 0.07, FDR = 0.01, *r* = 0.235; Fig. 8B), indicating that higher fecal melatonin levels are linked to a more stable and consolidated circadian rhythm as estimated by actimetry. This suggests that fecal melatonin could become gradually relevant to circadian maturation, with an effect emerging between 6 and 12 months in healthy infants.

In addition, exploratory analyses revealed positive associations between CFI and gut microbiome variables, including richness, Shannon diversity, and evenness (Supplementary Fig. 3A–D). These results show significant and positive associations between CFI and gut microbiome variables, highlighting the importance of CFI to broader changes in gut microbiome structure. These community-level associations are consistent with the notion that circadian rhythm consolidation is accompanied by broader changes in gut microbiome structure.

### Temporal stability of melatonin and time-based factors across two days

Next, we assessed the stability of the association between fecal melatonin and time-based factors by analyzing samples collected on two different days from the same infant using mixed-effect models. To quantify day-to-day variability, we applied an absolute difference approach. For example, if an infant’s fecal melatonin concentration was 150 pg/g on Day 1 and 160 pg/g on Day 2, the difference was calculated as *diff*_*melatonin* = *∣ melatonin_day1 - melatonin_day2∣*, which in this case equals 10 pg/g. Similarly, for *stool timing*, if collection occurred at 9:00 on Day 1 and 10:30 on Day 2, the absolute difference would be diff*_stool_timing* = *∣ time_day1−time_day2∣*, thus 1 h and 30 min. This approach was also applied to other time-based variables, including time since last stool, fasting time, and sleep pressure.

**Fig. 9:**
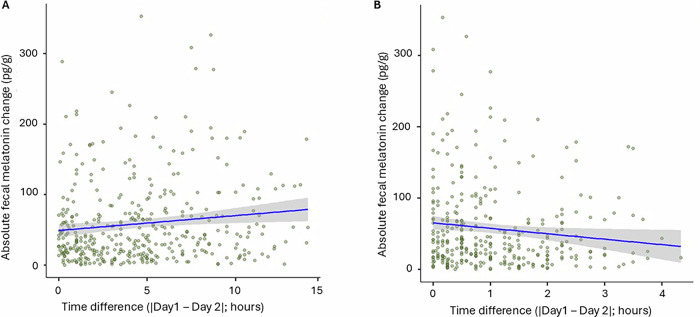
Temporal stability of fecal melatonin and its association with time-based factors across 2 days. **A** The relationship between the absolute difference in fecal melatonin concentration and the difference in *stool timing* (time of stool collection on Day 1 vs. Day 2; FDR = 0.011) shows a significant positive association, with greater discrepancies in stool timing corresponding to increased melatonin variability. **B** The relationship between the absolute difference in melatonin concentration and the difference in *fasting time* (time since last meal on Day 1 vs. Day 2; FDR = 0.017) shows a marginally significant negative association, with longer intervals between meals corresponding to reduced melatonin concentration.

A positive association was observed between the difference in *stool timing* and melatonin variability (coeff = 0.22, FDR = 0.011, *r* = 0.15), confirming that *stool timing* linked to fecal melatonin (Fig. 9). In contrast, a negative association was observed between the difference in fasting time and melatonin variability (coeff = −0.17, *r* = −0.13); however, this effect did not pass FDR correction (FDR = 0.077) (Fig. 9B). No association was observed neither for the variability in *time since last stool* (coeff = −0.07, *p* = 0.43, *r* = −0.065, −0.08) nor for *sleep pressure* with fecal melatonin (coeff= 0.03, *p* = 0.65, *r* = 0.034). Overall, these findings indicate that stool timing and, to a lesser extent, meal intervals are associated with fecal melatonin variability.

We then used separate linear models for each age group. At 3 months, a significant positive association emerged between *stool timing* differences and fecal melatonin across two days (coeff = 0.46, *p* = 0.008, *r* = 0.31). At 6 months, the positive association weakened (coeff = 0.23, p = 0.07, r = 0.16) and disappeared at 12 months. In contrast, at 3, 6, and 12 months, no significant associations were found between temporal stability of fecal melatonin and *time since last stool, sleep pressure, or fasting time*.

## Discussion

This study adds to understanding the role of intestinal melatonin in shaping early-life sleep and circadian rhythm. Using a longitudinal design, we quantified fecal melatonin and gut microbial composition in healthy infants, alongside sleep and circadian parameters obtained via continuous ankle-based actigraphy, 24-h diaries, and comprehensive surveys. From accelerometry data, a proxy for circadian maturation was computed (CFI), and diaries allowed for fine-resolution of time-based factors in relation to each stool sample (*stool timing, time since last stool*, s*leep pressure, fasting time*). This investigation provides several new insights: (1) Fecal melatonin increases with age but shows high inter-individual variability; (2) temporal factors influence melatonin dynamics, with lower levels in later stool collections and higher levels with more time passed since the last bowel movement; (3) higher fecal melatonin levels are associated with lower microbial richness and diversity; (4) fecal melatonin is correlated with specific ZOTUs, which decline with age, suggesting a dynamic but observational relationship between fecal melatonin and the gut microbiome; and (5) fecal melatonin is linked to circadian maturation, supporting a potential role in circadian system development; and (6) finally, *stool timing* variability and *fasting time* affect fecal melatonin stability, serving as potential regulatory mechanisms. Overall, these findings highlight an interplay between fecal melatonin, gut microbiota, and time-based factors in shaping early-life circadian and sleep-wake patterns, positioning fecal melatonin as a potential biomarker of circadian maturation.

The study first identifies the temporal regulation of fecal melatonin, with higher levels in stools collected earlier in the day at 3 and 6 months, an effect that diminished by 12 months. This suggests overnight melatonin accumulation in the gut, likely due to continuous local production and reduced excretion at night^12^. A positive association with *time since the last stool*, particularly at 6 months, indicates that longer intervals between bowel movements may lead to greater melatonin accumulation, linking gut-derived melatonin to intestinal motility^12^. In early infancy, when circadian rhythm and pineal gland activity remain immature, the gastrointestinal tract likely could thus play a major role in melatonin synthesis and regulation^31^. In contrast, no associations were found with *sleep pressure* or *fasting time*, possibly also affected by limited variability in *stool timing*. These findings suggest that the infant gut may act as a temporary reservoir of melatonin, and potentially a site of local melatonin production, with possible implications for neurodevelopment before pineal rhythmicity fully matures. Our analysis also underscores stool timing as an important contextual factor influencing fecal melatonin measurements, consistent with the idea that melatonin may accumulate in the gut lumen over time. Whether this reflects endogenous gut-derived meflatonin, passive accumulation, or a combination of both remains unclear. Additionally, breast milk melatonin, already known to support neonatal circadian entrainment^31^, and intestinal function^32^, likely contributes to fecal melatonin levels in early life. Future research should integrate simultaneous sampling of blood, stool, and breast milk, alongside controlled feeding and stool-collection timing, to disentangle the relative contributions of endogenous production, exogenous sources, and temporal factors.

The relationship between melatonin and the gut microbiome has gained attention due to its relevance to host physiology. In rodent models, intestinal melatonin modulates microbial composition and diversity^8^ and interacts with taxa, such as Bifidobacterium and Coprococcus, which are linked to metabolic pathways^33^. Melatonin also engages with microbial metabolites like short-chain fatty acids, supporting gut barrier function and circadian regulation^11^. Experimental studies further show that melatonin supplementation increases microbial diversity and shifts phylum-level composition in rodents^34^. In contrast, the infant cohort showed a negative association between fecal melatonin and microbial richness and diversity, especially at 12 months. This difference from rodent findings likely reflects the distinct study context: animal experiments use controlled melatonin exposure, whereas infant data capture naturally varying melatonin influenced by developmental stage, feeding patterns, and stool timing. As the infant gut and circadian system are still maturing, melatonin–microbiota relationships may function differently early in life. Interestingly, findings are consistent with rodent studies reporting no link between melatonin and overall relative abundance^35^, suggesting melatonin may influence specific microbial features rather than broad compositional changes.

The association between melatonin, ZOTUs and bacterial phyla is increasingly recognized as key to understanding gut microbiota. Melatonin supplementation enhances oxidative stress resistance and alters gut microbial composition in colitis models, increasing Firmicutes while reducing Bacteroidetes—a shift linked to a “healthier” microbial profile^36^. In rodents, melatonin-driven gut microbiota remodeling promoted genera like *Allobaculum*, *Bifidobacterium*, and *Faecalibaculum*, which produce short-chain fatty acids that mitigate oxidative stress and inflammation^37^. Despite these advancements, the relationship among systemic melatonin, ZOTUs, and bacterial phyla during early human life—a critical period for microbiome establishment and host-microbiota interactions—has received limited attention. Our study identified associations between fecal melatonin and 758 ZOTUs in infants. At 3 months, 333 ZOTUs were significantly linked to melatonin, decreasing to 163 by 12 months, suggesting that melatonin’s role (and potential influence) becomes more selective as the gut microbiota matures. Firmicutes emerged as the most dominantly associated phylum, which declined from 69.4% at 3 months to 46.6% at 12 months, consistent with findings in rodents^36^. In addition, *Actinobacteriota* and *Bacteroidota* gained relevance with age, becoming the second most associated phyla at infant age 12 months. These observations align with reports on gut-derived melatonin’s potential role in shaping microbial ecosystems, particularly taxa critical for gut health^12^. Interestingly, 85 ZOTUs negatively associated with melatonin were primarily from *Firmicutes*, *Proteobacteria*, and *Bacteroidota*, further supporting a potentially fine-tuned role of fecal melatonin in shaping the (infant) gut microbial community.

At the genus level, we observed age-related patterns in relation to fecal melatonin. The “Other” category remained dominant across all time points, highlighting the contribution of low-abundance or unclassified taxa. Notably, *Bifidobacterium* and *Veillonella* increased with age and were positively correlated with fecal melatonin. This relationship is observational, and future studies will be needed to determine whether and how melatonin might influence their metabolic pathways. In contrast, *Streptococcus* declined over time, and *Lactobacillus*, a genus with known immunomodulatory properties, was present a sighificant association at 3 and 6 months but did not exhibit significant associations with fecal melatonin at 12 months, possibly reflecting dietary transitions and microbial succession during late infancy^30^. Among ZOTUs negatively associated with fecal melatonin, Bacteroides and unclassified genera became more prominent with age, suggesting that elevated melatonin levels may suppress the early emergence of adult-associated taxa. These findings highlight an association between fecal melatonin levels and developmental changes in the infant gut microbiome, although the observational design does not allow conclusions about directionality or causality^37^.

Actimetry-derived sleep variables provide critical insights into sleep-wake maturation^38^. This study determined their association with fecal melatonin, offering a novel perspective on sleep rhythm emergence. Healthy sleep is fundamental for early brain and cognitive development^39^, with strong associations between sleep quality and cognitive function in young children^40^, lasting possibly way beyond^41,42^. Our findings reveal a weak link between fecal melatonin and sleep onset latency at 12 months. This aligns with research demonstrating that orally applied melatonin reduces sleep onset latency and increases total sleep duration in children with neurodevelopmental disorders^27,43^. Melatonin is widely used to address sleep problems in infants and children, particularly those with neurodevelopmental disorders, though its dosing and long-term safety remain uncertain^17,44^. Beyond synthetic supplementation, maternal melatonin in breast milk likely affects neonatal sleep-wake organization, as its fluctuations in composition have been linked to infant sleep disorders^45^. This relationship may also explain the here observed association between *fasting time* and fecal melatonin, reinforcing the concept that feeding patterns help entrain infant sleep, possibly through melatonin metabolism^46^. In alignment with this is the report that exogenous melatonin in night milk plays stabilizes sleep patterns in infants who have not yet developed endogenous melatonin production^16^. Our findings show an association between fecal melatonin levels and specific sleep parameters at 12 months, including sleep latency. These correlations may reflect parallel developmental processes or a direct role of melatonin in sleep–wake consolidation. Moreover, the association with sleep onset latency was detectable only at 12 months, with no significant relationship at earlier ages. This trajectory parallels observations in adults, where melatonin improves sleep quality by modulating specific sleep stages, such as non-rapid eye movement sleep N2^47^. In addition, we observed a weak association between higher fecal melatonin levels and longer sleep onset latency, particularly at 12 months. This finding may reflect inefficient metabolic utilization of melatonin, leading to increased excretion, or a compensatory increase in melatonin production in response to difficulties initiating sleep. Further research is needed to elucidate the underlying mechanisms and determine which of these interpretations is more plausible.

Our study uncovers a link between fecal melatonin levels and CFI at 12 months, pinpointing gut melatonin as possible orchestrator of systemic circadian rhythms. The relationship between systemic melatonin, e.g., saliva, and circadian regulation is well established. Salivary melatonin secretion patterns are temporally fine-tuned, with differences observed, for example, in dim light melatonin onset in children with autism spectrum disorder^19^. Accordingly, disruptions in melatonin timing significantly impact circadian parameters and activity rhythms. Guided through the central circadian clock, melatonin is an established hallmark of circadian rhythm disorders, neurodevelopmental conditions, and even neurodegenerative diseases, such as Alzheimer’s^48^, and thus established therapeutic potential in managing circadian dysregulation, inflammation, and neuroprotection^49,50^. While most studies focus on melatonin measured in saliva or blood^19,48–50^, our study examines a novel perspective with highlighting fecal melatonin as a candidate, population-level rhythm indicator in infancy. Our data reveal an association between fecal melatonin and CFI only in older infants (12 months), suggesting the link between fecal melatonin and circadian system maturation experiences a build-up period in infancy. Future research should explore whether fecal melatonin can be influenced through microbiota-targeted interventions, dietary modulation, or circadian-aligned care strategies. This could open new therapeutic avenues distinct from pineal melatonin pathways, particularly during early life when circadian systems in the midst of maturation.

Finally, this study reveals a connection between fecal melatonin variability and time-based factors, determining that stool timing (clock time) and meal intervals determine melatonin levels in infant stool. Meal timing has been shown to affect salivary melatonin rhythms in pregnant women, where temporal dietary intake assessed through a 3-day food record, was associated with variations in salivary melatonin levels^51^. Our data extend this concept to fecal melatonin in infants: the marginally significant negative relationship between day-to-day differences in fasting time and day-to-day variability in fecal melatonin suggests that larger differences in fasting duration are associated with reduced fluctuations in fecal melatonin levels. This stabilization may in turn support circadian maturation, as indicated by fecal melatonin association with CFI, highlighting the potential of feeding routines to shape early-life biological rhythms. In addition, antibiotic exposure was rare in this cohort, with no reported use at three months and only a small proportion thereafter (Table 1a). The absence of detailed post-enrollment antibiotic data remains a limitation and can be investigated in future studies.

Despite the novel insights provided by this study, important constraints must be considered. We did not account for variation due to dietary effects or due to maternal factors, such as antibiotic use or maternal sleep patterns. Further, our primarily cross-sectional stool sampling approach limited a precise tracking of intra-individual diurnal variations in melatonin, which we explored with data comparison among samples from two different days in the same subjects. Although we identified associations between fecal melatonin and sleep-wake or circadian measures, these relationships remain correlative, and their mechanistic link remains to be determined. A future question to disentangle in the future is the origin of fecal melatonin, whether derived from pineal secretion, enterochromaffin cells, or microbial synthesis. It remains exciting to be clarified to what extent fecal melatonin aligns with total gut melatonin or with systemic melatonin levels, underscoring the need for future studies with dynamic sampling, source-tracing techniques, and functional assays. Finally, future studies with larger cohorts should investigate potential sex differences in fecal melatonin dynamics and its interactions with the developing gut microbiome.

## Methods

### Participants

The study involved 184 healthy infants residing in Switzerland, ranging in age from 3 to 31 months, distributed across four sub-cohorts. These sub-cohorts were part of ongoing longitudinal Swiss infant research programs: SDEGU (Sleep Development and Gut Microbiota)^15^, focusing on early-life sleep regulation and microbial development; SPIN (Sleep Neurophysiology in Infancy)^52^, centered on infant neurophysiology assessments; NUTR (Early Nutrition and Infant Sleep), evaluating dietary and nutritional relationships on infant development; and SLEEPY (Infant Sleep Interventions)^53,54^, a cohort with sleep problematics, in collaboration with pediatricians. The primary ages of the longitudinal assessments were 3 months, 6 months, and 12 months for the SDEGU, SPIN, and NUTR cohorts of all types of sleepers, respectively. In comparison, the SLEEPY cohort included infants aged 5 to 31 months, focusing on parental reports of infant sleep problems. A subset of the population included in the current analysis was published earlier (SDEGU cohort, *n* = 152)^15^. Demographic information, including antibiotic use, feeding type, and gender distribution (Table 5), was available for the SDEGU, SPIN, and NUTR cohorts, representing approximately 92% of all samples, while such data were not available for SLEEPY.

**Table 5:** Participant demographics at 3, 6, and 12 months, including antibiotic use, feeding type, and gender distribution.

(a) Antibiotic use	Age 3 months	Age 6 months	Age 12 months
- Never	100%	97.2%	92.1%
- Reported intake	0%	2.8%	7.9%
(b) Feeding type			
- Exclusive breastfeeding	78.2%	42%	22.1%
- Exclusive formula feeding	0%	8.9%	51.7%
- Mixed feeding	21.8%	48.5	15.9%
- Others	0%	0.6	10.3%
(c) Gender distribution			
Male	53%	55%	55%
Female	47%	45%	45%

All participants were born vaginally at full-term, and were generally healthy. Further inclusion criteria were that infants were primarily breast fed at the time of inclusion, i.e., at least 50% of daily nutrition intake through breastfeeding at the first assessment, and parents were required to have a good knowledge of German or French language, see also^55^. Exclusion criteria were infants to have disorders central nervous system diorders, acute pediatric disorders, brain damage, chronic diseases, as well as family background of narcolepsy, psychosis, or bipolar disorder. Infants with birth weight below 2500 g, intake of medication affecting the sleep-wake cycle, or antibiotics prior to the first assessment were also excluded. Parents of the infants reported no history of gut-related disorders, such as irritable bowel syndrome, inflammatory bowel disease, or *Clostridioides difficile* infection, which could potentially affect the infants’ microbiome^15^. The study protocols were reviewed and approved by the Cantonal Ethics Committee of Zurich, Switzerland (BASEC 2016-00730, 2019-02250), ensuring compliance with the ethical principles outlined in the Declaration of Helsinki. Written informed consent was obtained from all primary parents/guardians after providing them with a comprehensive explanation of the study’s objectives and procedures.

### Experimental design and quantification of infant sleep variables

At each assessment timepoint (3, 6, and 12 months), continuous sleep data and at least one stool sample were collected per infant, yielding 423 parent-reported sleep data points (Brief Infant Sleep Questionnaire), 484 actimetry-based sleep measures (CFI), and 486 stool samples. Sleep data were obtained using ankle actimetry (GENEactiv accelerometer, Activinsights Ltd, Kimbolton, UK 43 × 40 × 13 mm, Micro-Electro-Mechanical Systems sensor, 16 g, 30 Hz frequency) combined with a paper-pencil 24-h sleep-wake diary. Parents documented actimeter removal in the diary, which was adapted after Werner et al.^56^. The diary included detailed 15-min interval reporting of sleep or wake periods, feeding, crying, and bedtimes. Using data from both actimetry and the 24-h diary, sleep and wake periods were identified following our laboratory’s established standards^57^. Further, demographic information and the Brief Infant Sleep Questionnaire (BISQ) were provided by parents in an online survey at each assessment^58^. Four key sleep variables from BISQ were selected for analysis: sleep latency, nighttime sleep duration, nighttime awakenings, and daytime sleep duration.

### A proxy for circadian rhythm: circadian function index

As in our recent work with infants, the Circadian Function Index (CFI) was calculated from ankle-worn actimetry data, including processing with MATLAB^22^. The CFI is a non-parametric index that quantifies the strength and regularity of circadian activity-rest patterns^22,59^. The CFI is a standardized computation based on three actigraphy measures, calculated using the “nparACT” package^60^: Intradaily Variability (IV), Interdaily Stability (IS), and Relative Amplitude (RA). Each measure is normalized to a scale from 0 to 1, and these values are combined to calculate the CFI using the formula:

1 $${CFI}=\frac{{IS}+\frac{(2-{IV})}{2}+{RA}\,}{3}$$

Specifically, IV measures the average frequency and degree of transitions between rest and activity within a day. Lower IV values indicate more consolidated and stable rest/activity periods, while higher IV values reflect frequent transitions or fragmented activity patterns. IS captures the consistency of activity patterns across days, with higher IS values representing a more stable and regular circadian rhythm across multiple days. RA reflects the contrast between peak activity and rest periods within a 24-h cycle, where higher RA values signify a more pronounced difference and stronger expression of rhythm. Thus, by integrating IS, IV, and RA, the CFI provides a comprehensive measure of circadian organization, where higher values indicate a more stable, well-defined rhythm and lower values reflect a fragmented or irregular pattern. For each infant, these three scores were averaged across days of continuous actigraphy recording, during which the ankle-worn device was worn 24 h per day, covering an average period of 9.16 ± 1.23 days across all ages (3, 6, and 12 months).

### Stool sample collection and processing

A total of 486 fecal samples were collected from 2020 to 2024 across the four independent cohorts: SDEGU (*n* = 410, from 152 infants: 129 at 3 months, 142 at 6 months, and 139 at 12 months), SPIN (n = 31, from 12 infants: 13 samples at 3 months and 18 at 6 months), NUTR (*n* = 21, from 8 infants: 9 samples at 3 months and 12 at 6 months), and SLEEPY (*n* = 24, from 12 infants ages 5-31 months, sampling age 12.2 ± 6.2 months, mean ± SD). Parents collected stool samples directly from diapers using disposable pipettes and spatulas, storing them in sterile tubes placed in protective bags for temporary refrigeration. Samples were transported to the laboratory within 72 h in cooling boxes, where they were aliquoted and stored at either −50 °C or −80 °C for subsequent analysis^15^. While 16S rRNA sequencing enables robust diversity analyses, it limits functional and strain-level inference. Gut microbiota data were obtained from the stool samples using 16s rRNA gene sequencing (details follow). Contextual information for each stool sample, including feeding, sleep-wake history, sample collection time, stool history, and further stool-related details were provided by parents or derived from the 24-h diary^15^. Gut microbiota profiling was performed using 16S rRNA gene sequencing as previously described^15^. Briefly, samples were processed in five sequencing batches, and batch was included as a covariate in all analyses to account for variability in sequencing depth. Samples with fewer than 50,000 reads (<1% of samples) were excluded, and the remaining data were rarefied to the minimum sequencing depth (50,268 reads), resulting in 1,430 bacterial amplicon sequence variants used for downstream analyses.

Microbial diversity, richness, and specific bacterial taxa (ZOTUs, i.e., zero-radius Operational Taxonomic Units) were analyzed using 16S rRNA sequencing. The temporal factors related to stool dynamics, derived from diaries and parent reports, included the time since the last bowel movement (*time since last stool*) and the clock time of stool passage (*stool timing*), alongside the time elapsed since the last sleep (*sleep pressure*) and time since the last meal (*fasting time*).

### Characterization of gut microbiota

DNA extraction from stool samples was performed using the PowerSoil DNA Isolation Kit (MOBIO Laboratories, Carlsbad, CA, USA), with approximately 200 mg of stool processed using minor modifications to the standard protocol. Following heat treatment (65 °C for 10 min, then 95 °C for 10 min), bacterial cells were lysed through bead-beating. The remaining steps adhered to the manufacturer’s instructions. 16S rRNA gene amplicons targeting the V3 region were generated using specific primers compatible with the Nextera Index Kit® (Illumina). Detailed procedures for amplification, barcoding, and sequencing are described in ref. ^15^. A comprehensive data processing and analysis pipeline was utilized to identify zOTUs via UNOISE^61^. Raw sequence data underwent quality checks with FastQC, followed by trimming, merging, and filtering with tools, such as seqtk, FLASH, and PrinSeq. Denoising and taxonomic assignment of amplicons were conducted using USEARCH, referencing the Greengenes database. Samples identified as outliers based on the interquartile range (IQR) were excluded. Core bacteria were defined as those present in at least 20% of samples with a relative abundance of 1% or more^21^.

Four key metrics were used to assess the gut microbiota composition: diversity, evenness, richness, and relative abundance. *Diversity*: Quantified as the Shannon index indicates the variety of species within the microbiome; higher values represent a more diverse community. *Evenness*: Reflects how evenly individual species are distributed within the community, with higher values indicating a more balanced distribution. *Richness*: Reflects the total number of distinct species present in a sample. *Relative abundance*: Determined by counting active zOTUs that exceeded a relative abundance threshold of 0.01, highlighting key contributors to microbial diversity in each sample. The analysis also included the relative abundance of major bacterial phyla—Proteobacteria, Actinobacteria, Firmicutes, and Bacteroidetes.

### Melatonin measurement

Fecal melatonin was quantified in 486 fecal samples (pg/g) using a radioimmunoassay (RIA), RK-MEL2 (NovoLytiX GmbH, Witterswil, Switzerland), following the manufacturer’s instructions (IFU, version 2022-03-07) with specific adaptations for stool samples. For extraction, 50 mg of stool was homogenized in 1 mL of RK-MEL2 Incubation Buffer, undergoing repeated vortexing and resting at room temperature to maximize homogenization. After centrifugation at 13,000 rpm (7000 × *g*) for 10 min, the supernatant was collected and centrifuged again to remove particulates before direct use in the RIA. A correction factor of 0.77 was applied to adjust for matrix effects, validated through spiking recovery experiments. The RIA protocol involved adding iodinated melatonin tracer and anti-melatonin antiserum to 0.4 mL of prepared stool samples, calibrators, and controls (run in duplicate). After incubation at 2–8 °C for 18–22 h, a solid-phase-bound secondary antibody was added to bind immune complexes, followed by a brief second incubation. Samples were then centrifuged, and the resulting pellets were analyzed using a gamma counter. Melatonin concentrations were determined using a 4-parameter logistic algorithm, with corrections applied for recovery rates. This assay had a sensitivity of 0.2 pg/mL (limit of detection) and 0.9 pg/mL (functional limit of quantitation). The average intra-assay and inter-assay precision were 8.2% and 11.7%, respectively.

### Statistical approach

Data processing, modeling, visualization, and statistical analyses were conducted using R version 4.2.1 along with the packages ggplot2 (https://ggplot2.tidyverse.org/), and MaAsLin2 (https://huttenhower.sph.harvard.edu/maaslin/) ^62^. MaAsLin2 was used to perform multivariate testing with mixed models, incorporating participant ID as a random effect to account for repeated measures within the same infant across different ages. Microbiota alpha-diversity metrics, including richness, Shannon diversity, and evenness, were computed using the *phyloseq* R package. For analyses within each age group, multivariate testing was conducted using linear models. This approach generated regression coefficients (coef), standard errors (std), *p*-values (*p*), and *q*-values (False Discovery Rate (FDR)), adjusted via the Benjamini-Hochberg procedure. This analysis examined a range of associations, including the association between stool melatonin and time-based factors: *time since last stool*, *stool timing*, *sleep pressure*, and *fasting time*. Furthermore, we examined the relationship between stool melatonin levels and gut microbiome composition—specifically the relative abundance of zOTUs, bacterial phyla, and genera—as well as age-related trends in all variables. Additionally, we evaluated links between melatonin and infant sleep (via sleep onset latency, nighttime sleep duration, night awakenings, and daytime sleep duration). The association between the CFI and fecal melatonin was also examined in this study. Finally, we assessed the stability of fecal melatonin with these time-based factors by inspecting the absolute difference between two samples from the same subject and timepoint collected on two different days (diff_melatonin = |melatonin_day1 – melatonin_day2|). Paired samples were collected on consecutive calendar days in most cases (a few with a 1- to 2-day gap, maximum interval 2 days), ensuring minimal developmental/dietary confounding. We set the significance level at *P* < 0.05 and applied a FDR correction to address issues of multiple comparisons. Fecal melatonin concentrations displayed a positively skewed distribution (skewness = +1.05); therefore, all associations were analyzed using generalized linear mixed-effects models implemented in MaAsLin2, which do not assume normality of the response variable and account for repeated measures within individuals. Finally, Per-group ZOTU-melatonin correlations were assessed using Pearson tests.

## Supplementary information

Supplementary information

## Data Availability

The data supporting the findings of this study are available at Figshare (https://figshare.com/s/4cf4fbaaa9315af0a19c).

## Code availability

The analysis code and scripts are accessible on GitHub: https://github.com/MNAl-Andoli/Melatonin-circadian-biomarker-gut-maturity.
