# Supplementary material for: Fecal melatonin as a biomarker of emerging circadian maturity and gut microbiota in infancy
Source: NPJ Biol Timing Sleep. 2026 Apr 28;3:17. doi: 10.1038/s44323-026-00080-6 (PMC13125696; doi:10.1038/s44323-026-00080-6)
Supplement: Supplementary file 1 — Supplementary information [file 44323_2026_80_MOESM1_ESM.pdf]

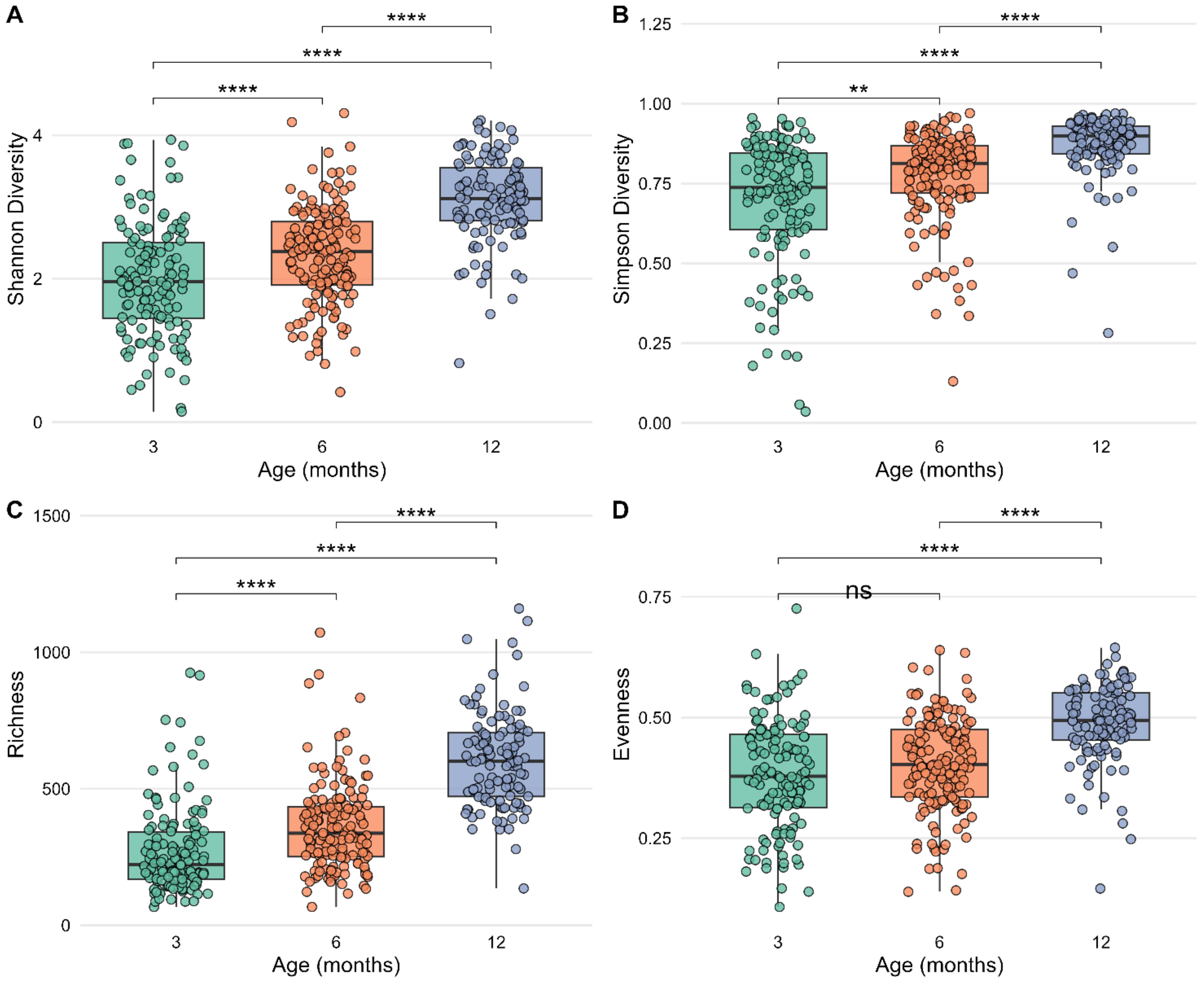

Supplementary Fig 1 | Age-related differences in gut microbiota alpha diversity during infancy.

Boxplots show **A** Shannon diversity, **B** Simpson diversity, **C** observed richness, and **D** evenness across infants at 3, 6, and 12 months of age. Kruskal–Wallis tests indicate significant overall differences across age groups for all alpha-diversity metrics ($p < 2.2 \times 10^{-16}$). Pairwise comparisons using Dunn's test with Bonferroni correction for multiple testing are indicated above each plot: **** $p < 0.0001$, ** $p < 0.01$ and "ns" indicates non-significant differences.

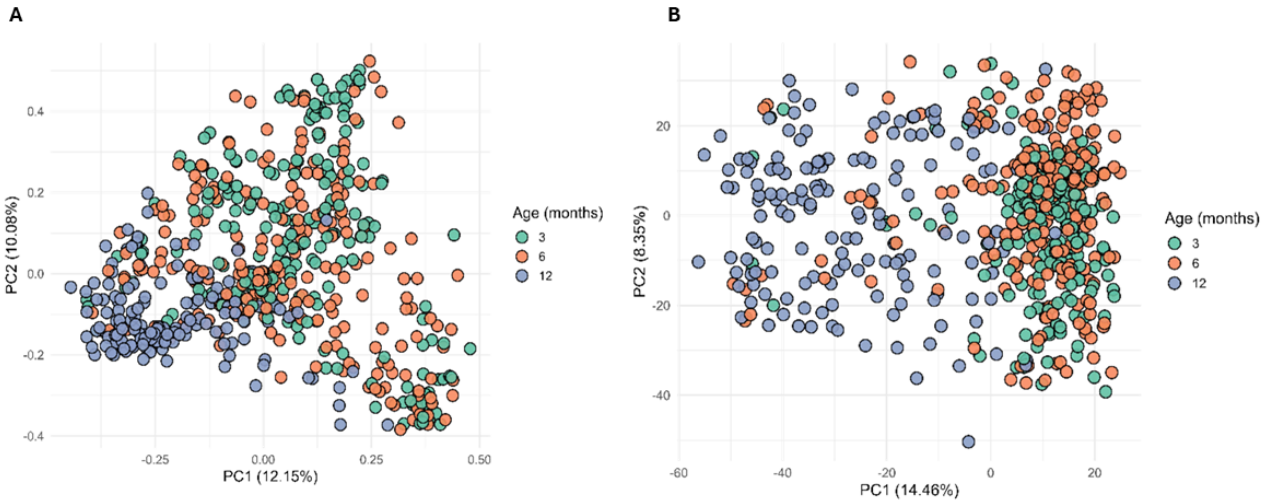

Supplementary Fig 2 | Age-related shifts in infant gut microbiome composition. PCoA ordination using **A** Bray–Curtis dissimilarity and **B** Aitchison distance (CLR-transformed) on ZOTU relative abundances. Both metrics show progressive community divergence with age, with clearer separation observed using the compositionally appropriate Aitchison distance.

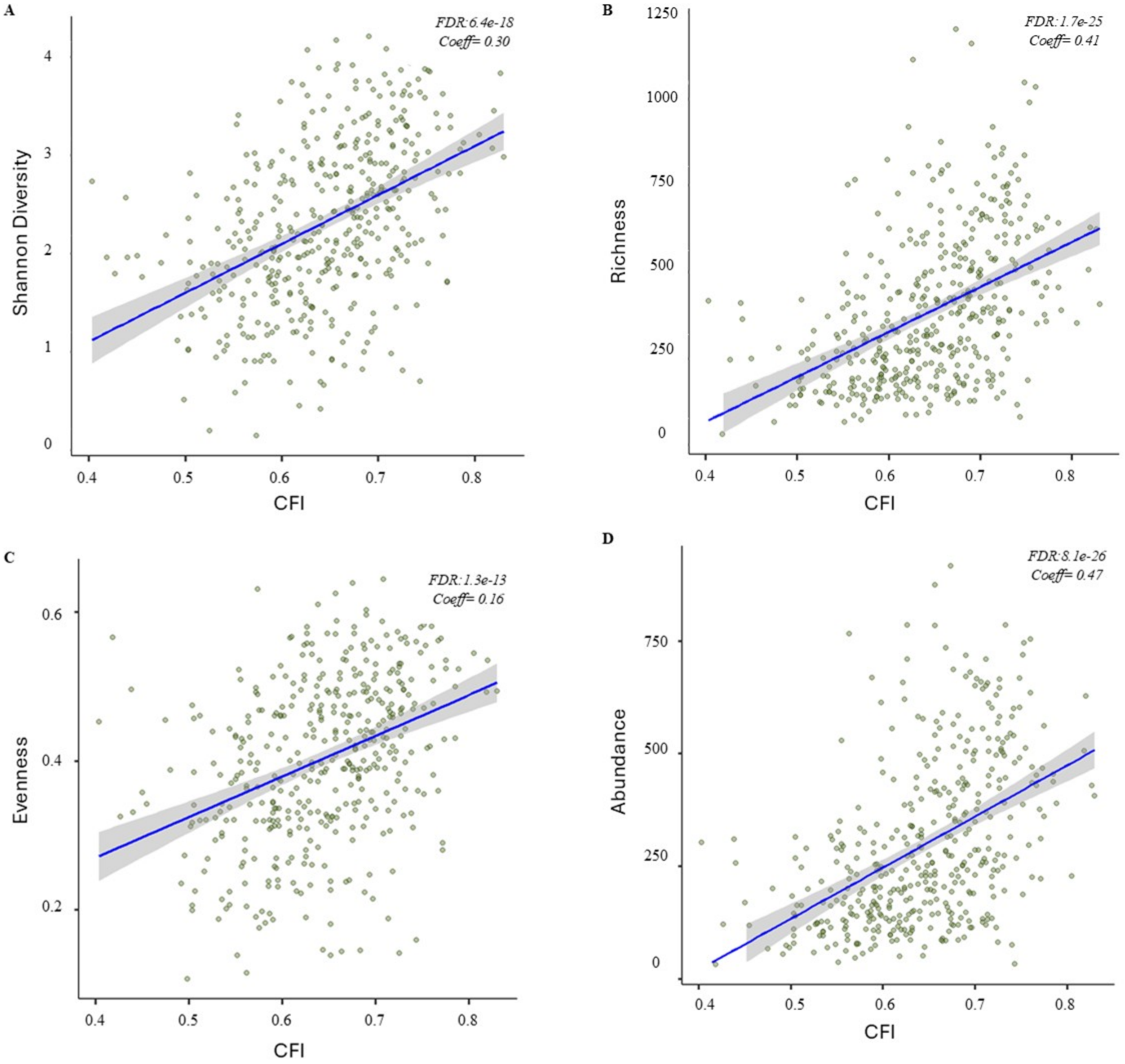

Supplementary Fig 3 | Associations between CFI and gut microbiome alpha diversity variables. Scatter plots show exploratory associations between CFI and **A** Shannon diversity, **B** richness, **C** evenness, and **D** overall microbial abundance. FDR–adjusted p-values and standardized coefficients show significant positive associations between CFI and all gut microbiome variables.
